# Dengue seroconversion among Israeli travelers to tropical countries.

**DOI:** 10.3201/eid0506.990615

**Published:** 1999

**Authors:** I Potasman, I Srugo, E Schwartz

**Affiliations:** Bnai Zion Medicla Center, Haifa, Israel. ipotasma@netvision.net.il

## Abstract

We tested for dengue seroconversion among 104 Israeli young adults who traveled to tropical countries for at least 3 months. Seven (6. 7%) seroconverted during travel; four (3.8%) had immunoglobulin (Ig) M antibodies; one was symptomatic with borderline IgM and a rise in IgG; two others (1.9%) had a rise in IgG titers, without detectable IgM. All four IgM-positive patients had traveled to Southeast Asia.


### Dengue Seroconversion among Israeli Travelers to Tropical Countries

**Israel Potasman,*† Isaac Srugo,*† Eli Schwartz‡**
*Bnai Zion Medical Center, Haifa, Israel; †Bruce Rappaport Faculty of Medicine Technion-Israel Institute of Technology, Haifa, Israel; ‡Center for Geographical Medicine, Sheba Medical Center, Tel Hashomer, Israel

We tested for dengue seroconversion among 104 Israeli young adults who traveled to tropical countries for at least 3 months. Seven (6.7%) seroconverted during travel; four (3.8%) had immunoglobulin (Ig) M antibodies; one was symptomatic with borderline IgM and a rise in IgG; two others (1.9%) had a rise in IgG titers, without detectable IgM. All four IgM-positive patients had traveled to Southeast Asia.

Dengue fever is a rapidly spreading mosquito-borne viral disease. Some 40% of the world's population lives in disease-endemic areas, and dengue outbreaks occur in more than 100 countries (1). Infected persons usually have high fever, chills, frontal headache, rash, severe myalgia, and malaise. Sometimes, the disease goes unrecognized (2).

According to surveillance data, nearly 200,000 cases of dengue fever occur in 31 countries in Central and South America (3); the attack rate among disease-endemic populations may be as high as 6,400 per 100,000 persons exposed. In contrast to data regarding the rate of dengue among populations in disease-endemic areas, data on the attack rate among travelers are scarce.

The rate of dengue fever has been examined in selected Japanese, Spanish, Swiss, and German travelers and in U.S. troops deployed in Somalia (4-8). The rate of dengue in these febrile patients was 6.9% to 65%. To the best of our knowledge, the rate of dengue fever has never been examined prospectively in a cohort of healthy, long-term travelers to disease-endemic areas.

### The Study

The study was approved by the Helsinki Committee of the Bnai Zion Medical Center, which provides service to approximately 1,500 travelers per year. Each traveler is requested to fill out a questionnaire including demographic, itinerary, and vaccination data, which are stored in a computerized database. The purpose of the study was explained to the travelers, and informed consent was obtained upon enrollment. Serum was drawn from random volunteers before starting the recommended vaccinations. Eligibility for inclusion was based on a minimum length of travel of at least 3 months and donation of a serum sample before and after travel. One hundred and four travelers fulfilled the inclusion criteria. The second serum sample was taken 1 to 4 months after returning home. Travelers who had positive dengue immunoglobulin (Ig) G serologic results were sent a questionnaire. In addition to demographics, the patients were asked to indicate their destination, season and length of stay, mosquito bites, use of repellents, fever, chills, nausea or vomiting, muscle aches, headache, cough, rash, or arthralgia. A case of dengue fever was defined according to Centers for Disease Control and Prevention (CDC) criteria (9). An asymptomatic dengue infection was one that met the laboratory criteria for diagnosis without clinical signs. Confidence intervals (CIs) were calculated with Statmate (GraphPad Software, San Diego, CA).

After thawing, 104 posttravel serum samples were tested for antibodies to dengue by an IgG enzyme-linked immunosorbent assay (ELISA) (Pan-Bio Pty, Ltd., Queensland, Australia). All positive sera were then tested in parallel with pretravel sera to confirm seroconversion. Seroconverting pairs were sent to the CDC laboratories in San Juan, Puerto Rico, for

Address for correspondence: Israel Potasman, Infectious Disease Unit, Bnai Zion Medical Center, P.O. Box 4940, 31048 Haifa, Israel; fax: 972-4-835-9755; e-mail: ipotasma@netvision.net.il.

confirmation and dengue IgM and IgG antibody determination.

IgM antibody was measured as a qualitative ELISA. An optical density of 0.2 or greater compared with a negative control was considered positive. The test may show some cross-reactivity to other flaviviruses (10). IgG antibody was measured as a quantitative ELISA using mixed dengue antigens. This test may also show cross-reactivity with other flaviviruses (11).

The mean age of the 104 study participants was 22.4 ± 2.2 years. The average length of stay abroad was 6.1 months (3 to 16 months), and the total time abroad for all 104 travelers was 53 person-years. The average time between serum samples was 11.1 months (4 to 22 months). The destinations were Southeast Asia (70%), South America (24%), Africa (4%), and both Southeast Asia and South America (2%).

Seven travelers (6.7%; 95% CI = 2.7-13.3%) had either an IgM antibody or a fourfold (or greater) rise in IgG titers after the trip. The median age of this group was 22 years, and four travelers were women. All seven stayed at their destinations (Table) during the summer months (some also spent the spring or autumn there). Their mean length of stay abroad was 5.3 months. The rates of conversion per month of exposure by continent were as follows: Southeast Asia, 5 (1.1%) of 451 (95% CI = 0.36-2.6%); South America, 1 (0.6%) of 159 (95% CI = 0.02-3.5%); and Africa, 1 (4%) of 25 (95% CI = 0.1-20.3%).

Four patients (3.8%) tested positive for dengue IgM after travel. All four had negative IgM dengue serologic test results before travel, and all but one had negative IgG titers. All four (two of them male) visited Southeast Asia, with Thailand being the only common destination. Two of these patients, who had traveled separately, indicated that they became ill on the island of Ko-Pangan. All four were engaged in extensive outdoor activities and consequently had mosquito exposure. All four used mosquito repellents (containing 20% to 25% DEET), but only three recalled being bitten by mosquitoes. Three travelers had fever, which in two (both female) was also accompanied by chills, headache, and protracted fatigue. One patient had an apparently asymptomatic infection.

Only two of the four IgM-positive patients had received Japanese B encephalitis vaccine after the initial blood collection, which could have interfered with after-travel testing. Of the three travelers who had a fourfold IgG rise, two were asymptomatic, and one had a clinical picture compatible with dengue fever 1 month after arriving in Thailand. This traveler also had a marginal IgM test. Among these three travelers, two had received yellow fever vaccine and one Japanese B encephalitis vaccine, which could have interfered with after-travel testing.

### Conclusions

Researchers have found that the younger the age, the higher the rate of illness among travelers, but dengue fever has not been thoroughly investigated (12,13). Dengue fever has not been reported in Israel over the last 50

Table. Dengue serologic results in seroconverting Israeli travelers to tropical countries

| Serum number | Age/sex | Destination | Length of stay (mos.) | Season of travel | Disease status | Results IgM | Results IgG |
|---|---|---|---|---|---|---|---|
| 4 | 52/F | Africa | 3 | Summer | Asymptomatic | Negative | 40 |
| 4a[a] | | | | | | Negative | 640 |
| 10 | 22/F | Southeast Asia | 6 | Spring-summer | Symptomatic | Negative | Negative |
| 10a | | | | | | +/- Pos.[b] | 160 |
| 11 | 21/F | Southeast Asia | 4 | Summer-autumn | Symptomatic | Negative | 40 |
| 11a | | | | | | Positive | 2,560 |
| 12 | 25/F | Southeast Asia | 3 | Summer-autumn | Symptomatic | Negative | Negative |
| 12a | | | | | | Positive | 160 |
| 13 | 26/M | Southeast Asia | 3 | Summer-autumn | Symptomatic | Negative | Negative |
| 13a | | | | | | Positive | 640 |
| 14 | 22/M | South America | 6 | Summer-autumn | Asymptomatic | Negative | 160 |
| 14a | | | | | | Negative | 640 |
| 18 | 21/M | Southeast Asia | 12 | Spring-summer | Asymptomatic | Negative | Negative |
| 18a | | | | | | Positive | 160 |

[a]a = after-travel sample.
[b]This test was marginally positive.

years, which made our group of young travelers particularly suitable for this study. In addition, there has been a dramatic increase in the number of Israeli travelers to tropical areas during the past decade. Approximately 40,000 Israelis travel to the tropics each year, more than 25,000 of whom are backpackers who travel for 3 to 12 months off the beaten track (14) and are exposed to the same diseases as travelers from other countries. Dengue fever has not been mentioned as a real hazard to Israeli travelers, despite an incidence in our study that may be as high as the rate of malaria without prophylaxis, and higher than the rates of hepatitis A, giardiasis, or typhoid fever (15). Nevertheless, it would be premature to extrapolate from our group of youngsters traveling on prolonged journeys, to groups with other travel characteristics. Older and perhaps short-term travelers may, for example, choose other tracks or adhere more closely to recommendations regarding insect repellents (16).

Four travelers (3.8%) of our group had IgM antibodies, indicating acute infection. Another traveler had symptoms of dengue fever with borderline IgM and a rising titer of IgG, which most probably reflected a recent infection. This traveler could have been infected earlier during her 6-month trip, and by the time the serum was taken, the IgM level might have dropped. Two additional travelers had a rise in IgG titers without detectable IgM.

Our results are tentative, as the serologic tests for dengue are not devoid of cross-reactivity (11). Both IgM and IgG may cross-react with other flaviviruses, such as Japanese B encephalitis, West Nile encephalitis, or yellow fever. The rate of IgG cross-reactivity between dengue infection and Japanese B encephalitis or yellow fever vaccine may be 17% to 40%; however, IgM cross-reactivity was not found after vaccination (E. Schwartz, pers. comm.). As none of our travelers had signs of encephalitis and yellow fever does not exist in Southeast Asia, the five travelers with IgM antibodies (including the one with a borderline case) contracted dengue fever. Indeed, four of them had clinical symptoms compatible with dengue fever. The diagnosis in the two travelers who had a fourfold rise in IgG titers was uncertain, since both were asymptomatic and had received yellow fever vaccines, which may have caused a cross-reaction in IgG assays. Dengue infection may be asymptomatic. Only three of the four IgM-positive patients were febrile; two of these also had chills. Asymptomatic dengue, which was found in three of our patients, has been described in populations of disease-endemic areas but never in travelers (2,17).

Two additional points in our study deserve comment. First, all four IgM-positive cases and the borderline IgM case occurred in travelers to Southeast Asia. A higher density of the vector and virus in Southeast Asia or visits by many of our travelers to Thai destinations—known for their high rate of dengue (18-20)—may have played a major role in this trend (our data are insufficient to permit conclusions regarding travel to Africa and South America). Secondly, all seven cases occurred during the summer, a season known for its high rate of mosquito activity and dengue transmission. The rate of dengue fever among travelers has been studied by four groups of researchers (4-7); an additional group described dengue in U.S. troops deployed in Somalia (8). The rate of dengue in their cohorts was 6.9% to 65%. However, all of these researchers focused on febrile patients with either fever of unknown origin, suspected dengue, or malaria. Hence, it is impossible to extrapolate from their data the actual risk of acquiring dengue during travel to the tropics.

Other travel clinics in Israel also have indicated that (after malaria) dengue is the second most frequent cause of hospitalization of returning travelers (20). Based on a minimum figure of the four IgM-positive patients, the calculated risk for dengue during a 1-month trip is thus 630 out of 100,000 travelers, which puts dengue high on the list of diseases contracted in the tropics. This statement holds true at least for Israeli travelers to the Far East. As patients may be evacuated because of dengue fever or may become sick after returning home, physicians need to be all the more vigilant in the face of this diagnostic possibility.

We conclude that dengue fever is perhaps the most common mosquito-borne disease of long-term young travelers, particularly those visiting Southeast Asia. The present results should serve as a further impetus toward the development of a vaccine for dengue fever.

### Acknowledgments

The authors thank Gary Clark and Vance Vorndam for performing the serologic tests and for their comments, and to R. Singer for excellent secretarial assistance.

This work was supported by the Research and Infrastructure Foundation, Israel Ministry of Health.

Dr. Potasman is head of Infectious Diseases at the Bnai Zion Medical Center, which is part of the Faculty of Medicine, Technion, Haifa. His area of expertise is travel medicine, with special interest in diseases among travelers, antimalarial drugs, vaccines, and *Helicobacter pylori*.

### References

1. World Health Organization/CTD. Dengue and DHF prevention and control. Geneva: The Organization; 1998.
2. Burke DS, Nisalak A, Johnson DE, Scott RM. A prospective study of dengue infections in Bangkok. Am J Trop Med Hyg 1988;38:172-80.
3. Lifson AR. Mosquitoes, models, and dengue [commentary]. Lancet 1996;347:1201-2.
4. Yabe S, Nakayama M, Yamada K, Kitano T, Arai Y, Horimoto T, et al. Laboratory virological diagnosis of imported dengue cases. Kansenshogaku Zasshi 1996;70:1160-9 (In Japanese).
5. Lopez-Velez R, Perez-Casa C, Vorndam A, Rigau J. Dengue in Spanish travelers returning from the tropics. Eur J Clin Microbiol Infect Dis 1996;15:823-6.
6. Settah SG, Vernazza PL, Morant R, Schultze D. Imported dengue fever in Switzerland—serological evidence for a hitherto unexpectedly high prevalence. Schweiz Med Wochenschr 1995;125:1673-8 (In German).
7. Jelinek T, Dobler G, Holscher M, Loscher T, Nothdurft H-D. Prevalence of infection with dengue virus among international travelers. Arch Intern Med 1997;157:2367-70.
8. Sharp TW, Wallace MR, Hayes CG, Sanchez JL, DeFraites RF, Arthur RR, et al. Dengue fever in U.S. troops during Operation Restore Hope, Somalia, 1992-1993. Am J Trop Med Hyg 1995;53:89-94.
9. Centers for Disease Control. Dengue fever. MMWR Morb Mortal Wkly Rep 1990;39:10-1.
10. Burke DS, Nisalak A, Ussery MA. Antibody capture immunoassay detection of Japanese encephalitis virus immunoglobulin M and G antibodies in cerebrospinal fluid. J Clin Microbiol 1982;16:1034-42.
11. Makino Y, Tadano M, Saito M, Maneekam N, Sittisombut N, Sirisanthana V, et al. Studies on serological cross-reaction in sequential flavivirus infections. Microbiol Immunol 1994;38:951-5.
12. Steffen R, Rickenbach M, Wilhelm U, Helminger A, Schar M. Health problems after travel to developing countries. J Infect Dis 1987;156:84-91.
13. Reid D, Grist NR, Najera R. Illness associated with "package tours": a combined Spanish-Scottish study. Bull World Health Organ 1978;56:117-22.
14. Berger SA, Giladi M, Shapira I. Health concerns of Israelis traveling to third world countries—experience of a travel advisory clinic. Harefuah 1994;126:410-2 (In Hebrew).
15. Reid D, Keystone J. Health risks abroad: general considerations. In: DuPont H, Steffen R, editors. Textbook of travel medicine and health. Vol 1. Hamilton, Canada: BC Decker; 1997. p. 3-9.
16. Fradin MS. Mosquitoes and mosquito repellents: a clinician's guide. Ann Intern Med 1998;128:931-40.
17. da Cunha RV, Dias M, Nogueira RM, Chagas N, Miagostovich MP, Schatzmayr HG. Secondary dengue infection in schoolchildren in a dengue endemic area in the state of Rio de Janeiro, Brazil. Rev Inst Med Trop Sao Paulo 1995;37:517-21.
18. Pick N, Potasman I. Dengue fever. Harefuah 1995;129:30-2 (In Hebrew).
19. Thavara U, Tawatsin A, Phan-Urai P, Ngamsuk W, Chansang C, Liu M, et al. Dengue vector mosquitos at a tourist attraction, Ko Samui, in 1995. Southeast Asian J Trop Med Public Health 1996;27:160-3.
20. Schwartz E, Mendelson E, Sidi Y. Dengue fever among travelers. Am J Med 1996;101:516-20.

